# A Gain-of-Function Germline Mutation in *Drosophila ras1* Affects Apoptosis and Cell Fate during Development

**DOI:** 10.1371/journal.pone.0023535

**Published:** 2011-08-12

**Authors:** Christopher Gafuik, Hermann Steller

**Affiliations:** Howard Hughes Medical Institute, The Rockefeller University, New York, New York, United States of America; University of Texas MD Anderson Cancer Center, United States of America

## Abstract

The RAS/MAPK signal transduction pathway is an intracellular signaling cascade that transmits environmental signals from activated receptor tyrosine kinases (RTKs) on the cell surface and other endomembranes to transcription factors in the nucleus, thereby linking extracellular stimuli to changes in gene expression. Largely as a consequence of its role in oncogenesis, RAS signaling has been the subject of intense research efforts for many years. More recently, it has been shown that milder perturbations in Ras signaling during embryogenesis also contribute to the etiology of a group of human diseases. Here we report the identification and characterization of the first gain-of-function germline mutation in *Drosophila ras1* (*ras85D*), the *Drosophila* homolog of human *K-ras, N-ras* and *H-ras*. A single amino acid substitution (R68Q) in the highly conserved switch II region of Ras causes a defective protein with reduced intrinsic GTPase activity, but with normal sensitivity to GAP stimulation. The *ras1^R68Q^* mutant is homozygous viable but causes various developmental defects associated with elevated Ras signaling, including cell fate changes and ectopic survival of cells in the nervous system. These biochemical and functional properties are reminiscent of germline Ras mutants found in patients afflicted with Noonan, Costello or cardio-facio-cutaneous syndromes. Finally, we used *ras1^R68Q^* to identify novel genes that interact with Ras and suppress cell death.

## Introduction

Multicellular organisms must extensively coordinate the activities of many diverse and highly specialized cells, requiring effective and flexible signaling mechanisms for both development and tissue homeostasis. Many inter-cellular signals are transmitted by receptor tyrosine kinases (RTKs), which control key aspects of cellular growth, differentiation, metabolism and cell death [1], [2]. On the other hand, mutations that lead to abnormal activation of RTKs can generate oncogenes that promote tumorigenesis [3], [4]. Ras proteins are guanine nucleotide binding proteins that act as molecular switches to transduce RTK-signals from the outside to the interior of the cell [5]. Remarkably, ∼20% of all tumors contain an activating point mutation in Ras [6], [7]. Consequently, this pathway has been extensively studied both in the context of normal signal transduction, and oncogenic growth. Because the RTK/RAS signaling network is highly conserved among animals, genetic model systems have made major contributions for elucidating this pathway [8], [9], [10], [11].

One major physiologic function of the RTK/RAS pathway during development is the transmission of anti-apoptotic signals that suppress the activation of an intrinsic cell death program [12], [13]. In *Drosophila*, as in mammals, cells are over-produced during development and compete for limiting amounts of extracellular survival factors in order to suppress the induction of apoptosis [14], [15]. This strategy permits appropriate matching of different cell types in a tissue and allows for the elimination of any superfluous and potentially dangerous cells. A conserved mechanism for survival signaling involves activation of receptor tyrosine kinases (RTKs) at the cell surface, which in turn stimulates the antiapoptotic activity of Ras [13]. Active Ras promotes its anti-apoptotic effect via several effector pathways, including the mitogen-activated protein kinase p42/44 (MAPK) of the ERK-type (extracellular signal-related kinase) via Raf [16], [17], [18], and the Akt kinase via Phosphoinositide 3-kinase [19]. In *Drosophila*, one major target for the anti-apoptotic activity of Ras is the pro-apoptotic Hid protein, which is inactivated via phosphorylation by MAPK [20], [21], [22]. Active Hid induces apoptosis by binding to and inhibiting *Drosophila* Inhibitor of Apoptosis Protein-1 (Diap1), an essential inhibitor of caspases in *Drosophila* [23], [24], [25], [26], [27]. In living cells, Diap1 inhibits both initiator and effector caspases, and its function is required for the survival of virtually all somatic cells [23], [25], [26], [28], [29], [30], [31]. In response to apoptotic stimuli, Diap1 is inactivated by natural IAP-antagonists, including Reaper, Hid and Grim (RHG proteins). The active forms of RHG proteins are generated in doomed cells by a combination of transcriptional induction and post-transcriptional regulation [32], [33], [34], [35]. Once active, RHG proteins form complexes that both disrupt binding of Diap1 to caspases and also stimulate auto-ubiquitination and degradation of Diap1, thereby removing the “brakes on death” [27], [36]. One important role of Hid is to recruit Reaper to the outer mitochondrial membrane, which is important for efficient inactivation Diap1 and apoptosis induction [27]. Survival signals, such as Spitz in the case of midline glia, inhibit the pro-apoptotic activity of Hid via activation of EGFR, Ras and MAPK, leading to direct phosphorylation of Hid by MAPK and inhibition of Hid pro-apoptotic activity [21], [33].

We previously conducted large-scale dominant modifier screens in *Drosophila* to identify genetic modifiers of Hid-induced apoptosis [20]. These screens identified several loss-of-function alleles in *sprouty* and *gap1,* both negative regulators of the RAS/MAPK signaling pathway and helped define the mechanism by which MAPK signaling inactivates a critical component of the apoptotic machinery [22], [37]. Here we report the identification and characterization of another Hid-modifier mutation, which maps to the switch II region of *ras1* (also known as *ras85D*), the *Drosophila* homologue of mammalian *N-ras, K-ras* and *H-ras*. Although many loss-of-function alleles have been described for *Drosophila ras1*, this mutation is the first endogenous gain-of-function allele reported for this gene. We demonstrate biochemically that this viable hypermorph, *ras1^R68Q^*, produces a defective Ras protein with reduced intrinsic GTPase activity, but normal sensitivity to GAP stimulation. These biochemical features are reminiscent of those recently described for mutant human H-ras and K-ras proteins known to underlie a group of related developmental disorders that includes Noonan syndrome, Costello syndrome and cardio-facio-cutaneous syndrome [38], [39], [40]. Flies mutant for *ras1^R68^* exhibit a number of developmental defects that are characteristic of abnormally elevated RTK/RAS/MAPK signaling, including enhanced resistance to apoptosis, supernumerary R7 cells in the eye and ectopic wing vein formation, demonstrating that the mutant Ras protein has enhanced signaling capacity *in vivo.* This allele should be a useful tool to study the physiological consequences of modest activation of Ras signaling *in vivo.* Finally, we used this mutant to identify novel interactors of Ras that suppressors cell death.

## Materials and Methods

### Fly stocks

The following fly stocks were used: *GMR-rpr^81^* [41], *GMR-rpr^34^* Cyo/Sco, *GMR-hid^1M^*, *GMR-hid^Ala3^* and *GMR-hid^Ala5^* [20], *GMR-hid^10^* and *hs-hid^3^* [24], *GMR-grim* [42], *GMR-phyl* [43], *GMR-rho^1^* [44], *vg-GAL4* (F.M. Hoffmann, unpublished), *UAS-hid* [45], *arg^IΔ7^* [46], EGFR^−^  =  *flb^f2^* [47], *rl^10a^* [48], *sev-ras1^N17^* [49], P[*slit-1.0-lacZ*] [50], *Hml-GAL4, 2xUAS-EGFP* (J.A. Rodriguez, unpublished). Stocks for meiotic recombination mapping (*ru^1^ h^1^ th^1^ st^1^ cu^1^ sr^1^ e^s^ ca^1^* and *ru^1^ h^1^ th^1^ st^1^ cu^1^ sr^1^ e^s^ Pr^1^ ca^1^*/TM6B, *Bri^1^, Tb^1^*) and stocks for P-element induced male recombination mapping (y^1^ w*; CyO, H{PDelta2-3}HoP2.1/Bc^1^ Egfr^E1^ as a source of transposase and all P-element insertion lines) were obtained from the Bloomington Stock Center (Bloomington, IN). All other lines were generated by meiotic recombination of the appropriate alleles.

### Genetic screens

Dominant modifier screens were performed as described in Fig. S1. Approximately 170,000 F1 progeny from ENU and EMS mutagenized *GMR-rpr^81^* flies were screened for modification of a *GMR-rpr^81^* induced rough eye phenotype, yielding 25 enhancers and 5 suppressors (Table S1). Similarly, 300.000 F1 progeny from ENU, EMS and x-ray mutagenized flies were screened for suppression of a *GMR-hid^10^* induced rough eye phenotype, resulting in the recovery of 128 additional suppressors (Table S2). In sum total, 158 dominant modifiers of *GMR-rpr* or *GMR-hid* were isolated in these screens.

Complementation analyses using phenotype and map information placed 133 of these modifiers into 13 complementation groups, while the remaining mutants represent single hits or have no recessive phenotype. To further enrich for mutants that are cell death specific, we eliminated general modulators of GMR promoter expression or eye development by testing modifiers against *GMR-phyl* and *GMR-rho* induced rough eye phenotypes, which are unrelated to cell death [43], [44]. In addition, reasoning that mutants involving apoptosis genes should be able to modify cell death phenotypes in contexts other than the eye, suppressors from the *GMR-hid* screen were tested for their ability to suppress *hs-hid* induced embryonic lethality and *vg-GAL4*, *UAS-hid* induced wing ablation. On the basis of these secondary screens, we eliminated several complementation groups including *glass*, which encodes the transcription factor that drives GMR expression, *Su(GMR)2A* and *su(GMR)3A*, which are known to indirectly and non-specifically affect GMR promoter expression, and *Su(GMR-hid)3A* and *Su(GMR-hid)3B*, complementation groups that have not been assigned to a previously characterized gene [51], [52]. We also eliminated 4 alleles linked to the parental *GMR-rpr* transgene. Our cell death enriched subset of modifiers therefore consists of 58 mutants in total, 40 that fall into 6 complementation groups and 18 single alleles.

All crosses and suppression experiments were carried out at 25°C except crosses with *vg-GAL4* and *UAS-hid*, which were performed at both 18°C and 25°C. Suppression experiments with hs-*hid* were done by heat shocking 1^st^ instar larvae at 37°C for 15 minutes.

### Biochemistry

A cDNA clone encoding *Drosophila ras1* was obtained from the *Drosophila* Genomics Resource Center (clone ID: RE53955) and the entire *ras1* ORF was subcloned into pBluescript (Stratagene). Mutant *ras1^R68Q^* was generated using the QuikChange II XL Site-Directed Mutagenesis Kit (Stratagene). The Ras ORFs were then subcloned into pET-28a (Novagen) in frame for an N-terminal His tag. Catalytic human p120-Gap (GAP-285, amino acids 714–998, IMAGE Clone: 4829173, Open Biosystems) was subcloned into the pET41a vector (Novagen) to generate an N-terminal GST tag. Fusion proteins were expressed in BL21(DE3) *E*. coli (Invitrogen) and affinity purified on an AKTA Purifier (Pharmacia) using a HisTrap FF column (GE Healthcare) for Ras proteins and a GSTrap FF column (GE Healthcare) for GAP-285. Ras purification was done according to the procedure described for human H-Ras [53]. GAP-285 was expressed by inducing cells for 16 hours at 30°C with 0.2 mM IPTG.

Intrinsic GTPase activities were measured using [gamma-^33^P]GTP (3000 Ci/mmol, NEN) and the EasyRad Phosphate Assay (Cytoskeleton) [54]. GAP-stimulated GTPase activities were measured with a real-time assay using the fluorescent substrate MDCC-PBP (Invitrogen) and 2 *µ*M Ras protein, with or without, 0.02 *µ*M GAP-285 [55].

### Phenotypic Analyses

To visualize larval hemocytes, wandering 3^rd^ instar larva expressing *UAS-EGFP* driven by *Hml*-GAL4 were collected and immobilized on ice prior to imaging [56]. MG cells in stage 17 embryos were visualized using P[*slit-1.0-lacZ*] and ß-gal immunohistochemistry as previously described [57]. The number of MG was averaged for segments T2 to A5. Tangential sections (1 *µ*m) of adult eyes were prepared according to standard protocols for analysis of ommatidia [58].

## Results

### Genetic screens for dominant modifiers of apoptosis in *Drosophila*

Dominant modifier screens are designed to detect pathway components for which small perturbations in gene dosage can alter a sensitized phenotype, thus allowing for the recovery of both loss-of-function and gain-of-function mutations. We used eye-specific expression of the *Drosophila* cell death genes *hid* or *rpr* under control of the GMR promoter to generate a dosage sensitive eye ablation phenotype and then screened for dominant modifiers of this phenotype to identify regulatory components of the intrinsic cell death program [20], [24], [41]. Although several genes identified in this way have been reported, details of these screens have not been previously published and are provided in the supplementary material (Fig. S1, Tables S1 and S2).

We identified a subset of 58 mutants that specifically affect *rpr* or *hid*-induced cell death. 40 of these correspond to 6 complementation groups, while 18 represent single alleles. Of the 6 complementation groups identified, 3 correspond to genes that regulate EGF receptor (EGFR) signaling. Five loss-of-function alleles each of *gap1* and *sprouty*, both negative regulators of EGFR/MAPK signaling, were recovered as strong, *hid* specific suppressors. These mutants have been characterized elsewhere [20]. We also isolated five loss-of-function *Star* alleles as enhancers of *GMR-rpr*. *Star* is required for the correct processing of Spitz, a stimulatory ligand of EGFR [59]. Ten alleles of *diap1,* the major biochemical target for the pro-apoptotic activity of RHG proteins were isolated, including loss-of-function alleles that enhance *rpr*-, *hid*- and *grim*-induced cell death and two distinct classes of gain-of-function alleles [23], [28], [31]. The fifth complementation group, consisting of 12 alleles, displayed a differential modulation of cell death phenotypes reminiscent of *diap1* RING mutants and was found to encode *dbruce*, the *Drosophila* ortholog of mouse Bruce and human Apollon [60], [61]. This very large (4852 amino acid) BIR-containing protein is cytoprotective against caspases and required for spermatid survival [62], [63], [64]. Finally, 5 single alleles from the *GMR-rpr* screen likely represent weak hypomorphs of *diap1* as they map close to the *diap1* locus, and a sixth allele was identified as an allele of *Delta*.

The remaining complementation group, *Su(GMR-hid)2A*, and 12 additional single alleles were previously not characterized. We chose one allele, *Su(21-3s)* for further analysis based on its ability to potently suppress *hid*-induced phenotypes (eye/wing/organismal lethality) without non-specifically affecting *GMR-phyl* (Table S2). *Su(21-3s)* mutants are homozygous viable and do not have overt abnormalities. This mutant was mapped by meiotic recombination to the right arm of chromosome 3, near the visible marker *curled.*

### Characterization of *Su(21-3s)* suppressor phenotypes

**Figure 1:**
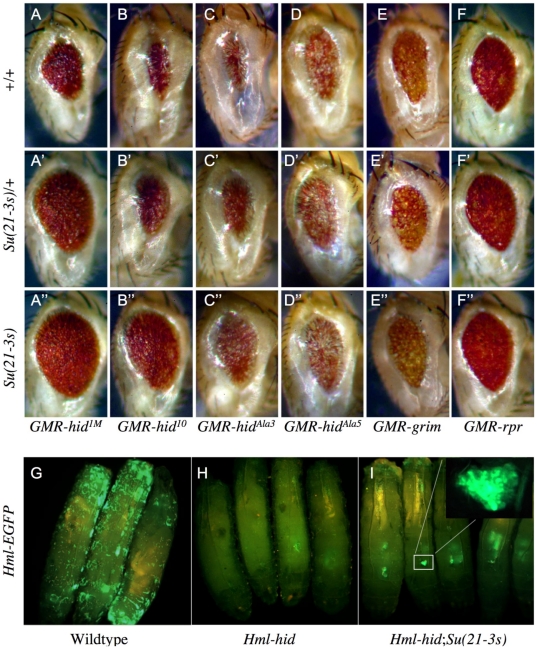
Suppression phenotypes of *Su(21-3s)*. *GMR*-*hid* but not *GMR*-*grim* or *GMR*-*rpr* induced cell death is dominantly suppressed by *Su(21-3s)* in a manner that requires intact MAPK phosphorylation sites in the overexpressed Hid protein. (A–F) The resulting rough eye phenotype is strongly suppressed in a dosage dependent manner by one (') or two (″) copies of the *Su(21-3s)* mutation when induced by overexpression of either a weak, *GMR-hid^1M^* (A) or a strong, *GMR-hid^10^* (B) allele of *GMR*-*hid*, but is only weakly attenuated by *Su(21-3s)* when induced by *GMR-grim* (E) or *GMR-rpr* (F). In addition, *Su(21-3s)* suppresses cell death induced by overexpression of a Hid protein lacking 3 of 5 predicted MAPK phosphorylation sites, *GMR-hid^Ala3^* (C) but not by *GMR-hid^Ala5^* (D), a *hid* allele lacking all 5 MAPK consensus sites (Bergmann, et al. 1998). (G-I) Death of larval hemocytes induced by overexpression of Hid under control of the hemocyte specific driver Hml is also partially suppressed by the *Su(21-3s)* mutation. (G) EGFP is used to visualize hemocytes in wildtype 3^rd^ instar larva: *Hml-GAL4*, 2x*UAS-EGFP.* (H) Overexpression of Hid in hemocytes results in their complete ablation by the 1^st^ instar larval stage: *Hml-Gal4*, 2x*UAS-EGFP*; *UAS-hid.* (I) *Su(21-3s)* is able to partially suppress hemocyte death induced by Hid. Surviving hemocytes appear to be concentrated within the lymph glands as shown in the inset: *Hml-Gal4*, 2x*UAS-EGFP*; *UAS-hid*, *Su(21-3s)*.

We first examined more rigorously the suppression phenotypes of *Su(21-3s)* in the eye by testing the modifier effects of one or two copies of *Su(21-3s)* against various GMR expression constructs. This analysis confirmed that *Su(21-3s)* potently suppresses *GMR-hid* induced cell death in a dosage dependent manner (Fig. 1A,B). We found suppression of *GMR-rpr* and *GMR-grim* phenotypes, however, to be extremely weak, even with two copies of *Su(21-3s)* (Fig. 1E,F). Given that *hid* is highly expressed in the developing eye, we believe the small effect exerted by *Su(21-3s)* on *GMR-rpr* and *GMR-grim* is due primarily to a suppression of endogenous Hid activity and not that of Rpr or Grim [24]. We conclude from these data that *Su(21-3s)* is a specific suppressor of *hid* induced cell death.

The activity of Hid is regulated by the EGFR/MAPK pathway in a manner that depends on intact MAPK phosphorylation sites in Hid. Our analysis here reveals that *Su(21-3s)* readily suppresses *GMR-hid^Ala3^*, a *hid* allele lacking 3 of 5 predicted MAPK phosphorylation sites, but fails to suppress *GMR-hid^Ala5^*, which is missing all 5 MAPK sites (Fig. 1C,D) [37]. This requirement for one or two of the predicted MAPK phosphorylation sites in Hid (Ser-121 and Thr-228), along with the observed specificity for *GMR-hid* suppression, strongly suggested that *Su(21-3s)* might be mediating its suppressive effects through the EGFR/MAPK pathway.

We further extended analysis of the *Su(21-3s)* suppression phenotypes to the developmental context of larval hemocytes, an important model system for the study of vertebrate haematopoiesis [65], [66]. *Drosophila* hemocytes require trophic signaling from multiple pathways for their survival and in its absence undergo caspase dependent cell death [67], [68]. Larval hemocytes also undergo caspase dependent cell death in response to ectopic *hid* expression [69]. Using a hemocyte specific promoter to drive expression of *UAS-EGFP*, we are readily able to visualize hemocytes in wandering 3^rd^ instar larvae (Fig. 1G) [56]. Ectopically expressing *UAS-hid* using the same driver results in near complete ablation of hemocytes by the 1^st^ instar larval stage (data not shown) and generates 3^rd^ instar larvae that are completely devoid of hemocytes (Fig. 1H). We found that the *Su(21-3s)* mutation is able to partially suppress this cell death such that EGFP expressing hemocytes are clearly visible anteriorly in the lymph glands of 3^rd^ instar larvae (Fig. 1I). Circulating hemocytes, however, appear to remain susceptible to *hid*-induced cell death and are missing, even in the presence of two copies of the *Su(21-3s)* allele. It may be that *Su(21-3s)* is a weak suppressor of cell death in hemocytes, sufficient to suppress Hid activity in young hemocytes localized to the supportive environment of a lymph gland, but insufficient in the context of a mature circulating hemocyte.

### *Su(21-3s)* is a viable gain-of-function allele of *ras1 (ras85D)*

**Figure 2:**
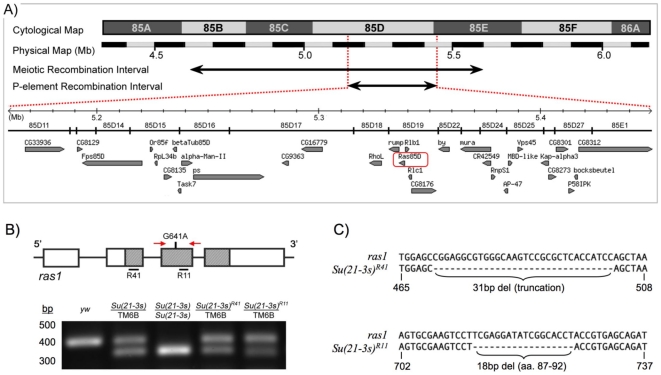
*Su(21-3s)* is a gain of function allele of *ras1* (*ras85D*), the *Drosophila* homolog of human *n-ras*, *h-ras* and *k-ras*. (**A**) The cell death suppression phenotype of *Su(21-3s)* mutants was localized by meiotic recombination to the right arm of the 3rd chromosome as indicated by the large horizontal arrow. P-element induced male recombination mapping further localized this suppressor to the region depicted by the short arrow. An enlargement of this interval (5.162-5.452 Mb on the physical map) is shown below, illustrating the ORFs contained therein. The *ras85D* (*ras1*) locus, outlined with a red box, was sequenced in a candidate gene approach and a G to A transition in exon3 (G641A) was identified. (**B,C**) A screen for reversion of the dominant *Su(21-3s)* suppressor phenotype generated a number of genetic revertants, two of which, *Su(21-3s)^R11^* and *Su(21-3s)^41^,* were molecularly determined to be intragenic loss of function *ras1* alleles. (**B**) A schematic of the *ras1* locus, with exons boxed and coding regions stippled, illustrates the *Su(21-3s)* point mutation in exon 3 (G641A) and the small intragenic deletions identified in *Su(21-3s)^R11^* and *Su(21-3s)^R41^* (labeled R11 and R41 respectively, with deleted sequences underlined in black.) The red arrows correspond to primers used in a PCR diagnostic (below) used to confirm that both revertants contain the original G641A mutation. (**C**) Sequence analysis of these lethal revertants using strand specific PCR revealed that *Su(21-3s)^R11^* contains an 18bp deletion that removes essential amino acids 87–92 from the Ras1 protein. *Su(21-3s)^R41^* was found to have a 31bp deletion, resulting in a frameshift that generates a truncated Ras1 protein.

**Figure 3:**
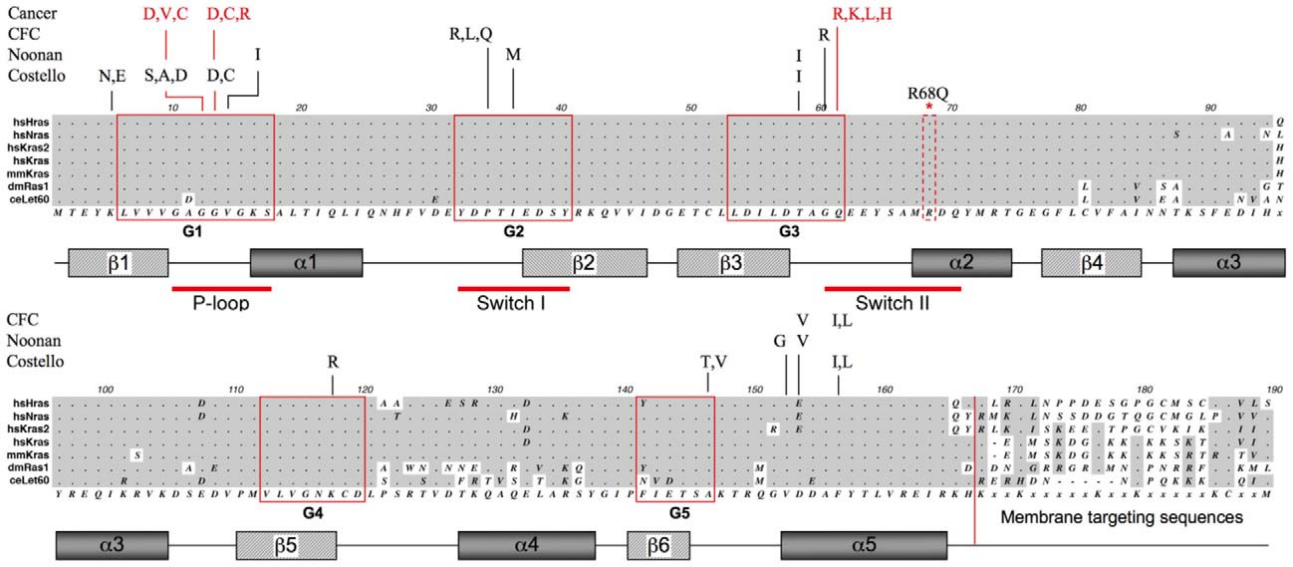
Amino acid alignment of fly, worm and mammalian Ras1. These homologs have extensive primary sequence homology. *Drosophila* Ras1 (dmRas1), for example, is 87% identical to human K-ras (hsKras) at the amino acid level when C-terminal membrane-targeting sequences are excluded. Conserved regions are shaded in grey, with residues identical to the consensus sequence represented by a grey dot, while non-conserved residues remain unshaded. Five highly conserved signature motifs named “G box” sequences, labeled G1-G5 and boxed in red, are found in all families of small GTPases. Secondary structural elements are depicted as rectangles below the primary sequence alignment (alpha-helices, α1-α5, are dark grey and ß-sheets, ß1–ß6, light grey) and the phosphate-binding loop (P-loop), which binds the γamma-phosphate of GTP, and the nucleotide-sensitive switchI and II regions are indicated. The Switch regions are known to undergo large conformational changes upon exchange of bound GDP for GTP. The mutational spectrum of Ras is illustrated above the alignment, showing the distribution of amino acid substitutions encoded by germline mutations found for the developmental disorders Noonan, Costello and CFC syndromes and the most frequent cancer-associated somatic mutations (labeled in red). R68Q indicates the mutation characterized in this study, a non-conserved arginine to glutamine amino acid substitution within the switch II region of *Drosophila* Ras1 (dashed red box). hs, *H. sapiens*; dm, *D. melanogaster*; mm, *M. musculus*; ce, *C. elegans.*

In order to identify the gene responsible for the *Su(21-3s)* phenotype, we mapped it by a second, finer round of meiotic recombination to a 1 Mb interval between 85A and 85E, then further localized the mutation by P-element mediated male recombination to a 270 Kb interval between 85D11 and 85E1 (Fig. 2A). Given that *Su(21-3s)* differentially suppresses *hid*, but not *grim* or *rpr* in a manner reminiscent of EGFR/MAPK pathway mutants, we suspected that *Su(21-3s)* might be a rare hypermorphic allele of *ras1,* or *ras85D* as it is otherwise known, because it is located within this interval. Indeed, when we sequenced *ras1* in a candidate gene approach, a G to A transition in exon3 was identified. This mutation results in an amino acid substitution at position 68 of the Ras protein, replacing a positively charged arginine within the universally conserved switch II region of Ras with a neutral glutamine (Fig. 3).

The switch regions of Ras have been defined as regions that undergo a large conformational change when Ras transitions from the GTP- to the GDP-bound state [70]. Detailed biochemical analysis and crystal structures have revealed that residues in the switch II region of Ras contact and are stabilized by the GTPase Activating Protein (GAP), allowing them to participate up to a 1000 times more efficiently in the catalysis of GTP [71]. As a consequence, mutations in the switch II region of Ras interfere with its catalytic GTPase activity and prolong the time Ras remains bound to GTP. Mutations that reduce the GTPase activity of Ras are hypermorphic since it is the GTP-bound form of Ras that engages and activates downstream signaling effectors. The signaling activities of Ras are terminated when GTP bound by Ras is converted to GDP, explaining why the most frequently occurring oncogenic mutations in Ras, at amino acids 12,13 and 61, also render Ras biochemically inert as a GTPase (Fig. 3) [72], [73]. It seemed feasible, therefore, that the R68Q mutation identified in *Su(21-3s)* flies could similarly result in a Ras protein with reduced GTPase activity, leading to a prolonged RAS/MAPK signal that suppresses cell death induced by *GMR-hid*.

We reasoned that if the *Su(21-3s)* phenotype is due to a gain-of-function mutation in *ras1*, it should be revertible by introduction of a second, intragenic loss-of-function mutation. To test this, we conducted a reversion screen for loss of the *Su(21-3s)* suppression phenotype and successfully recovered two mutants containing intragenic loss-of-function *ras1* mutations (Figs. 2 and S1). One revertant contains a 31 bp deletion in *ras1* that results in a Ras protein truncated at amino acid 87. The second revertant contains an in frame 18 bp deletion of *ras1* that removes amino acids 87–92 which are essential for Ras function [74]. These revertants have genetic properties of *ras1* null alleles and fail to complement the known null alleles *ras1^e1B^* and *ras1^e2F^* and supporting the idea that the *Su(21-3s)* phenotype is due to a revertible gain-of-function mutation in *ras1*.

As an allele of *ras1*, *Su(21-3s)* should interact genetically with other members of the MAPK signaling pathway in a predictable manner. We crossed *GMR-hid^10^* flies in a *Su(21-3s)* background to mutants of MAPK signaling and observed the degree of cell death in the eye (Fig. S2). MAPK signaling mutants tested include *argos, ras1, rolled* and *EGFR*. In this analysis, we found that the *Su(21-3s)* mutant behaves in a manner consistent with that expected for a gain-of-function *ras1* allele. For example, *Su(21-3s)* is not much affected by loss-of-function mutations in upstream components of MAPK signaling, such as *argos* or *EGFR* but is strongly ameliorated by loss of downstream components, such as *rolled*. Additionally, when a dominant negative form of Ras1 (*sev-ras1^N17^*) is expressed in the eye, the suppressive effects of *Su(21-3s)* are completely abrogated. On the basis of our mapping data, the reversion screen, the sequence data and the genetic interaction data presented above, we conclude that *Su(21-3s)* is a hypermorphic allele of *ras1,* which we rename here, *ras1^R68Q^*.

### Biochemical analysis of recombinant Ras1^R68Q^ protein

**Figure 4:**
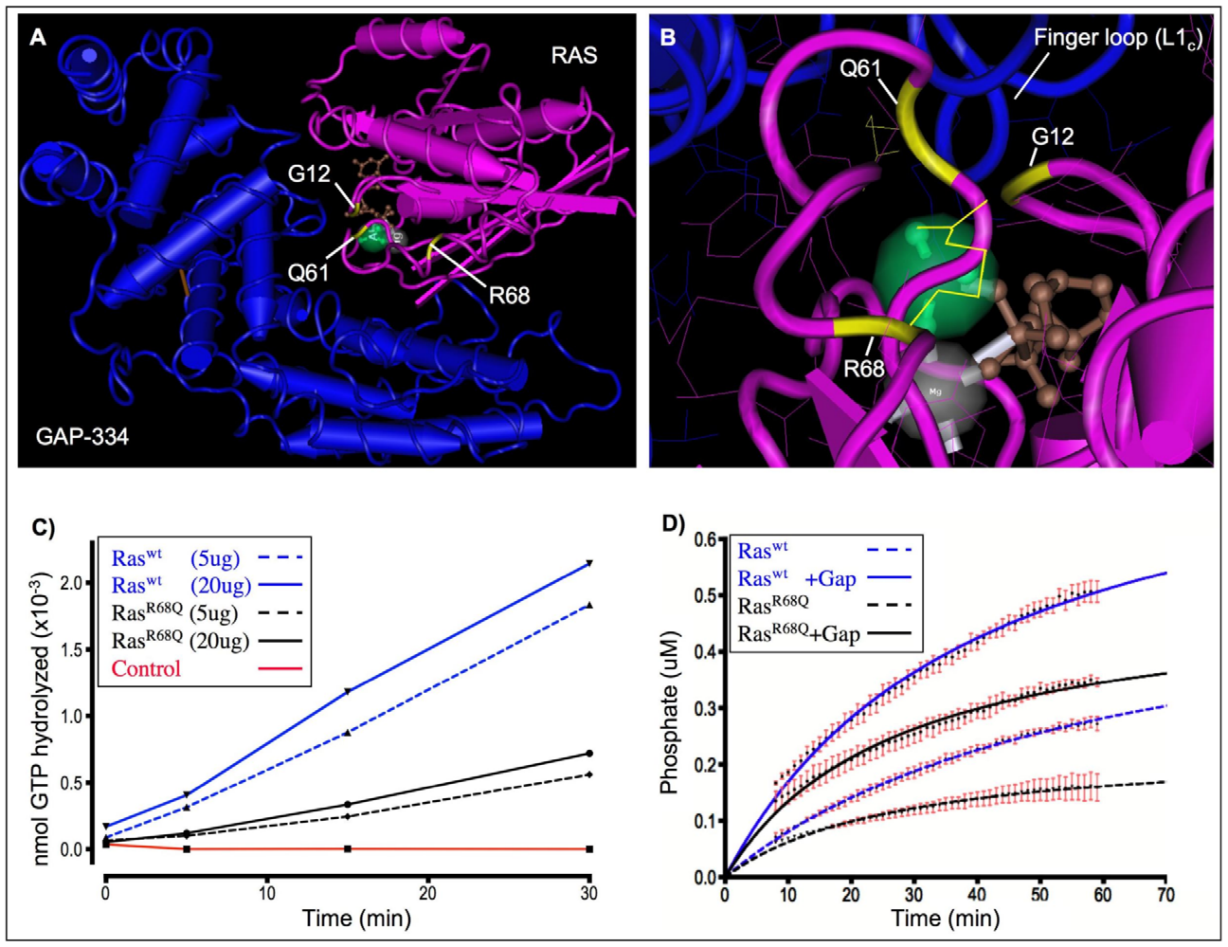
Structural and biochemical analysis of wildtype and mutant Ras1. (A–B) Three-dimensional crystal structure of human H-Ras (pink) bound to the GTPase-activating domain of human GTPase-activating protein p120^GAP^ (GAP-334, blue) in the presence of aluminum fluoride (AlF_3_, green.) The positions of oncogenic residues glycine-12 (G12) and glutamine-61 (Q61) as well as the mutant residue in *ras1^R68Q^* flies, arginine-68 (R68), are shown in yellow. The Switch II region of Ras, of which Q61 and R68 are a part, is stabilized by GAP-334. (B) An enlargement of (A) showing the finger loop of GAP-334, which supplies an arginine side chain (arginine-789) into the active site of Ras to neutralize developing charges in the transition state (Scheffzek et al., 1997). R68, located proximally to the catalytic site of Ras, also extends a positively charged guanidinium group towards the active site. The images were constructed using the Entrez software Cn3D with mmdbId:51925 (Chen et al., 2003). Guanosine diphosphate (GDP,brown); Mg^2+^ (grey). (C) The intrinsic GTPase activities of affinity purified drosophila Ras1^wt^ (blue) and Ras1^R68Q^ (black) were determined using a kinetic phosphate assay employing [γamma-^33^P]GTP as a substrate. The conditions of the assay are such that the reaction proceeds with unimolecular kinetics and is insensitive to the amount of Ras protein employed (dashed vs. undashed lines). The mutant Ras1^R68Q^ has an intrinsic GTPase activity that is approximately 1/3 that of wildtype Ras1 (*k_cat_* = 0.020 min^−1^ and 0.063 min^−1^ respectively.) (D) Human GAP-285 protein was purified by affinity chromatography and its ability to stimulate wildtype and mutant *Drosophila* Ras1 proteins was tested using a real-time fluorescent assay. Both wildtype and mutant Ras1 proteins are sensitive to GAP stimulation (dashed vs. undashed lines). Data is the average of three independent experiments. Error bars are in red.

To test the hypothesis that exchanging a positively charged arginine with a neutral glutamine at position 68 of Ras results in a protein with deficient GTPase activity, the intrinsic GTPase rates of wildtype and mutant *Drosophila* Ras1 proteins were measured. Full-length wildtype Ras1 and mutant Ras1^R68Q^ were bacterially expressed and purified as His-tagged fusion proteins, yielding large amounts of pure, catalytically active enzyme. Intrinsic GTPase activity rates were measured with a kinetic phosphate assay employing [γamma-^33^P]GTP as substrate. This sensitive assay revealed that Ras1^R68Q^ has an intrinsic GTPase activity that is approximately 1/3 that of wildtype Ras1, with enzymatic rates (*k_cat_*) of 0.020 min^−1^ and 0.063 min^−1^, respectively (Fig. 4C). Since many activating Ras mutations also result in an enzyme that is insensitive to GTPase activating proteins (GAPs), the ability of Ras1^R68Q^ to be stimulated by GAP was also assessed. Recombinant human GAP-285 protein was purified by affinity chromatography and its ability to stimulate wildtype and mutant Ras1 proteins was tested using a real-time fluorescent assay. These experimental data show that Ras1^R68Q^ remains amenable to GAP stimulation (Fig. 4D). This means that in contrast to constitutively active Ras mutants such as oncogenic Ras^V12^, whose GTPase activity is completely refractory to stimulation by GAPs, Ras1^R68Q^ can be regulated and is able to cycle between on and off states [75]. Together, these biochemical data support the hypothesis that Ras1^R68Q^ has a reduced basal level of GTPase activity, remains in its active GTP-bound form for longer and thus has an enhanced signaling capacity, but is still amenable to regulation, making it compatible with nearly normal cellular function and organismal development. These biochemical features are highly reminiscent of those recently described for germline H-ras and K-ras mutants found in the developmental disorders Noonan syndrome, Costello syndrome and cardio-facio-cutaneous syndrome [38], [40].

### *Ras1^R68Q^* promotes survival of midline glia (MG)

**Figure 5:**
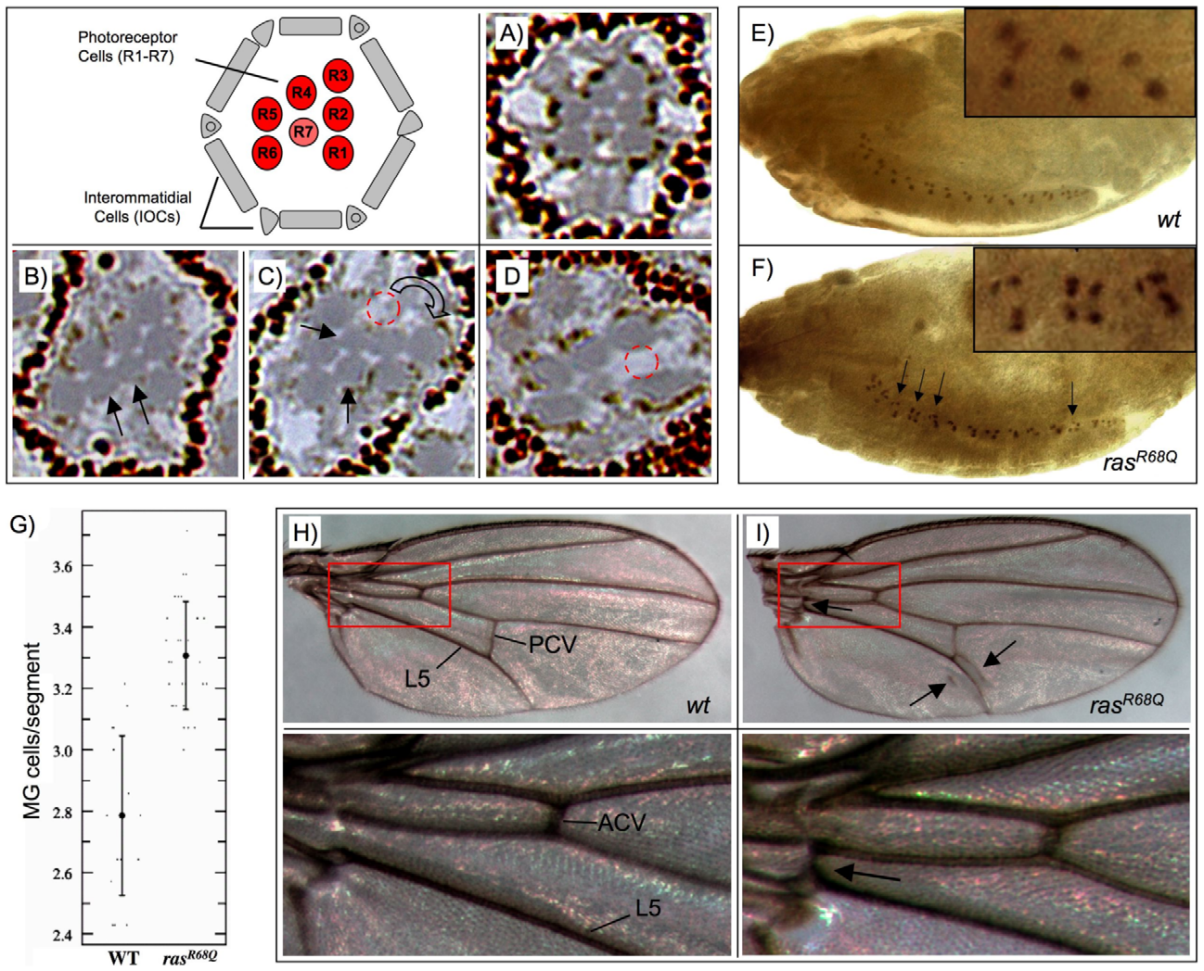
*Ras1^R68Q^* mutants exhibit several developmental defects characteristic of elevated Ras/MAPK signaling. (**A–D**) Semi-thin plastic sections of adult eyes were prepared and analyzed for defects in ommatidia formation. (**A**) Wildtype ommatidia are precisely ordered, containing one R7 cell and six outer photoreceptor cells. (**B–D**) *Ras1^R68Q^* ommatidia show two defects typical of mutations that increase Ras/MAPK signaling during eye development; supernumery R7 cells (arrows, B,C) and mislocalized (red circle, C) or missing (red circle, D) outer photoreceptor cells. The schematic illustrates the major cell types present in ommatidia. (**E-G**) Midline glial (MG) cells were visualized in wildtype (**E**) and *ras1^R68Q^* (**F**) stage 17 embryos using the MG-specific reporter construct P[*slit-1.0-lacZ*]. During development, the majority of MG undergo apoptosis such that at this stage only about three MG per segment normally survive. Elevated Ras/MAPK signaling allows for increased survival of MG cells and is reflected by an increase in the number of persisting MG cells per segment (arrows and inset, F). (**G**) Wildtype embryos contain an average of 2.8 MG cells per segment (n = 448) whereas *ras1^R68Q^* embryos contain an average of 3.3 MG cells per segment (n = 420). This difference is statistically significant by an unpaired t-test (p_95_≤0.0001). (**H,I**) Flies bearing the *ras1^R68Q^* allele develop ectopic wing material including extra longitudinal ‘veinlets’ near the posterior crossvein and an extra crossvein near the wing hinge (arrows, I) The area boxed in red is shown magnified below. PCV, posterior crossvein; ACV, anterior crossvein; L5, L5 wing vein.

The survival of *Drosophila* midline glia (MG) cells during embryonic development depends on survival signals mediated by the EGFR/Ras/MAPK pathway [21], [76]. During formation of the *Drosophila* central nervous system, there are initially approximately ten MG cells per segment at stage 13. Most of these undergo apoptosis in a RHG-dependent manner such that by stage 17, only three MG per segment survive [45], [77]. We tested the effect of *ras1^R68Q^* in this system. MG cells were visualized in wildtype and *ras1^R68Q^* embryos using the MG-specific *pslit-lacZ* reporter, and marked MG cells were carefully counted. This analysis revealed an increase in the number of MG cells in *ras1^R68Q^* embryos as compared to wildtype embryos (Fig. 5E,F). Stage 17 wildtype embryos contained an average of 2.8 MG cells per segment (n = 448) whereas *ras1^R68Q^* embryos contained an average of 3.3 MG cells per segment (n = 420). This difference is statistically significant by an unpaired t-test (p_95_≤0.0001) and is consistent with an increase of Ras activity in *ras1^R68Q^* flies (Fig. 5G).

### *Ras1^R68Q^* causes supernumerary R7 cells in the eye

The adult *Drosophila* eye comprises about 800 ommatidia, each with a precise, reproducible structure consisting of eight photoreceptors and 12 accessory cells [78], [79]. Adoption of a neuronal cell fate by the precursor of the R7 photoreceptor requires an inductive signal from the neighboring R8 cell and is dependent on EGFR/MAPK signaling [80], [81], [82]. Furthermore, the cone cell precursors are capable of acquiring an R7 cell fate if MAPK signaling is ectopically activated in these cells, resulting in extra R7 cells that are easily visualized [83]. To determine if the *ras1^R68Q^* mutation exerts effects in a paradigm other than apoptosis, semi-thin plastic sections of adult eyes were prepared and analyzed for defects in ommatidia formation. We observed two types of defect in *ras1^R68Q^* flies typical for mutations that increase RAS/MAPK signaling during eye development. First, we detected ommatidia with supernumery R7 cells (Fig. 5B,C). Second, we noticed occasional ommatidia missing outer photoreceptor cells, also a phenotypic consequence of elevated MAPK signaling (Fig. 5D) [83]. The developmental defects in retinal cell differentiation observed here further supports our hypothesis that *ras1^R68Q^* is a gain-of-function *ras1* allele.

### The wings of *ras1^R68Q^* flies contain ectopic vein material

In addition to defects in the eye and midline glial cells, *ras1^R68Q^* flies also show abnormalities in adult wing tissues. Homozygous *ras1^R68Q^* flies have an extra longitudinal “veinlet” branching off the posterior crossvein (Fig. 5I). Additionally, an ectopic longitudinal vein was seen beneath the posterior crossvein and an ectopic crossvein appeared between the L4 and L5 wing veins near the hinge. These defects are remarkably similar to those observed in the wings of *rl^sem^* and *DER^Ellipse^* flies, which have elevated levels of MAPK signaling [84]. When *UAS-ras1^R68Q^* was overexpressed in the wing using *en-Gal4,* extensive ectopic wing vein material and blisters developed (Fig. S3). Overexpression of wildtype Ras1 gave a similar but less severe phenotype. Finally, we attempted to express *ras1^V12^* in the wing using *en-Gal4* but found this to cause organismal lethality.

### Overexpression of *ras1^R68Q^* in the eye induces both severe overgrowth and cell death

It was previously shown that overexpression of wildtype Ras1 in the *Drosophila* eye, even at the high levels obtained by transgene expression, results in little or no observable phenotypic effect [83]. For this reason, studies of elevated Ras signaling in *Drosophila* regularly rely on a very strong, constitutively active Ras^V12^ mutant allele. We similarly observed that wild-type *UAS-ras1* expressed by *GMR-Gal4* had little effect on eye development in 11 independent transgenic lines (Fig. S4). In striking contrast, seven independent transgenic lines expressing *UAS-ras1^R68Q^* resulted in highly distorted eyes that exhibited both hyperplastic tissue overgrowth and widespread cell death ablation phenotypes. For purposes of comparison, we attempted to express two different *UAS-ras1^V12^* alleles in the same manner, but again found this induced organismal lethality (likely due to the fact that *GMR* drives some expression in tissues other than the eye and Ras1^V12^ induces non-cell autonomous cell death when overexpressed) [85]. We generated many more than seven *UAS-ras1^R68Q^* transgenic lines but similarly found many of them to be lethal in combination with *GMR-Gal4*. This lethality was not observed in any of the 16 independent *UAS-ras1* transgenic lines tested. These experiments further support the view that Ras1^R68Q^ is an activated form of Ras that nevertheless remains amenable to regulation and therefore is less biologically potent than the constitutively active Ras1^V12^ protein.

### Identification of Novel Ras Interactors and Suppressors of Cell Death

In the reversion screen described above we were also able to recover additional suppressors of *GMR-hid*. We collected a number of strong dominant suppressors of the *GMR-hid* eye phenotype and mapped them using deletions on the 3^rd^ chromosome. As indicated in Table 1, we successfully recovered 14 suppressors that fall into 8 complementation groups. In most cases, we were able to identify a single gene that appears to be responsible for the suppressors phenotype (indicated in bold). In two cases, the mutations were narrowed to a small region, but we were unable to unequivocally identify a single candidate. We also recovered mutations in the *glass* gene, which affects expression from the GMR-driver [86]. We recovered mutations in Gap1 and Delta, both of which were identified in our original *hid* suppressor screen, indicating an overlap in the mutational spectrum between the two screens [20]. Based on previous reports, mutations in Gap1 are expected to suppress GMR-hid, and Delta/Notch signaling is known to intersect and cooperate with the Ras/Mapk pathway [87]. Interestingly, we also isolated a number of novel *ras* interactors, including four alleles of the predicted transcription elongation factor Su(Tpl) and an allele of notum, a component of the Wnt/Wingless signaling pathway. These results indicate that use of the *ras^R68Q^* allele in screens may indeed uncover novel regulatory interactions that have been missed with other strategies, including those that make use of the constitutively active, non-regulatable *ras^V12^* hypermorph.

**Table 1:** Suppressors of *GMR-hid* recovered from the reversion screen.

Complementation Group	Location	Candidate Gene
SupX3/SupX6	67C10	***Gap1***
SupE6.1/SupE6.2	66E6-67B1	*Argk*?
SupE8.1/SupE8.2	66B6-66C1	*ERR*?
SupX9/SupE10.1/SupE10.2/SupX13	76D3	***Su(Tpl)***
SupX8	72C3	***notum***
SupE7.2	99E4	***hdc***
rasR68Q interactor1	62B1	***drpr***
rasR68Q interactor2	92A1	***Dl***

In most cases, a single mutant gene corresponding to these suppressors could be identified (indicated in bold). In two cases, the mutations were narrowed to a small region but a single gene could not be unequivocally identified; in these cases, we list the most likely candidate gene (with an “?”) based on mapping data and published literature.

## Discussion

We have conducted genetic screens for dominant modifiers of cell death induced by the *Drosophila* IAP-antagonists, *hid* and *rpr*. From over 150 mutants initially isolated, secondary screens allowed us to identify 58 cell death specific modifiers. Of these, 40 alleles were placed into six complementation groups that define both known and unknown genes. These include *Star*, *gap* and *sprouty* involved in EGFR/MAPK signaling, the known cell death regulator *diap1*, the very large BIR and UBC containing *dbruce*, and an unknown gene, *Su(GMRhid)2A* that remains unidentified. Here we focused on a previously uncharacterized cell death suppressor originally termed *Su(21-3s).* Using a combination of meiotic and P-element induced male recombination, genetic reversion, biochemistry and *in vivo* analysis, we demonstrate that this mutant is a gain-of-function mutation in *ras1* (*ras85D*), the *Drosophila* homolog of human *K-ras, N-ras* and *H-ras*. We also show that this allele affects cell fate decisions and the pattern of normal, developmental apoptosis in paradigms known to depend on Ras-signaling.

One important role of Ras signaling during development is the transmission of an anti-apoptotic signal [13], [20], [22]. As previously reported, the pro-apoptotic protein Hid contains 5 potential MAPK phosphorylation sites that are essential for its sensitivity to Ras-mediated inhibition [20]. A Hid protein with either 3/5 or 5/5 mutant MAPK sites (Hid^Ala3^ and Hid^Ala5^, respectively) was refractory to suppression by the gain-of-function MAPK allele *rl^Se^* ^m^ (a very mild suppression by *rl^Sem^* is due to phosphorylation of the endogenous wildype Hid protein). In contrast, there was still some suppression of Hid^Ala3^ and Hid^Ala5^ by RasV12. It was postulated that this might be due to the ability of Ras, unlike MAPK, to exert additional anti-apoptotic effects through activation of the PI3-K/Akt-kinase effector branch. In the current study, we found that *ras^R68Q^* was able to partially suppress Hid^Ala3^ but not Hid^Ala5^ (Fig. 1C,D). Because Hid^Ala3^ retains two phosphorylation sites, it appears that partial phosphorylation of Hid is sufficient for a mild inhibitory effect, and that all five phospho-acceptor sites need to be eliminated in order for Hid to become refractory to inhibition by MAPK. Furthermore, it appears that Ras^R68Q^, unlike Ras^V12^, is unable to exert an additional suppressive effect via PI3-K/Akt-kinase. Perhaps the enhanced signaling activity of Ras^R68Q^ is able to activate the MAPK effector branch, but does not reach a required threshold to engage the PI3-K/Akt-kinase pathway [59], [88], [89], [90], [91]. This may also help to explain the organismal viability of Ras^R68Q^ as compared to Ras^V12^. Along the same line, Ras^R68Q^ was able to suppress Hid-induced cell death of lymphocytes within the protective environs of the lymph gland but not of those that were circulating (Fig. 1G-I). In sharp contrast, over-expression of Ras^v12^ in hemocytes not only leads to survival of circulating hemocytes but in fact results in a massive overproliferation of hemocytes (Rodriguez and Steller, unpubl. data) These results serve to highlight the exquisite sensitivity of biological systems to the degree of Ras signaling and suggest that between the extremes of wildtype Ras and constitutively active Ras^V12^ lies a large spectrum of biological responsiveness.

Ras is highly conserved among metazoans and a number of Ras structures have been published that make it possible predict how mutations in specific regions might affect function. In the case of Ras^R68Q^, we considered that this change may affect the transition state of Ras. According to the “arginine-finger hypothesis” proposed by Scheffzek and colleagues, GTPase-activating-proteins (GAPs) dramatically accelerate the GTPase reaction of Ras by supplying an arginine side chain (arginine-789 in the case of GAP-334) into the active site of Ras to neutralize developing charges in the transition state [71]. A detailed analysis of the interactions between Ras and GAP-334 showed no role for R68 of Ras, explaining why Ras^R68Q^ can be stimulated by GAP [71], [74], [92], [93]. However, a close inspection of the Ras catalytic site (Fig. 4B) shows that R68 extends its side chain towards the catalytic center [94]. Mutating R68 to glutamine removes a stabilizing positive charge from the transition state and, according to the arginine-finger hypothesis, would be expected to result in less efficient hydrolysis of GTP. We tested this prediction biochemically and indeed found that Ras^R68Q^ hydrolyzes GTP intrinsically at a reduced rate, approximately 30% of that of wild type GTP (Fig. 4C.)

Oncogenic mutations in Ras occur most frequently at codons 12,13 or 61 and result in an enzyme with deficient GTPase activity. This renders Ras inactive because Ras is ‘on’ when bound to GTP and switches ‘off’ by hydrolyzing bound GTP to GDP. Inhibition of Ras GTPase activity therefore stabilizes Ras in its active conformation, prolonging its recruitment and activation of downstream signaling components [5], [7], [10], [70], [95]. The reduced GTPase activity of Ras^R68Q^ means that it would remain in its active GTP-bound conformation for longer periods of time allowing for enhanced signaling to downstream effector pathways. As noted above, however, Ras^R68Q^ may not remain in an active state sufficiently long to engage the catalytic p110 subunit of PI3K. An interesting alternative possibility however may be that R68 is directly involved in an interaction with PI3K and a mutation in R68 negatively affects this interaction. This raises the intriguing possibility that some of the phenotypes described for Ras^R68Q^ (Fig. 5) may actually be due to a loss, rather than a gain of PI3K activity.

During the initial mapping and characterization of *ras1^R68Q^*, we conducted a reversion screen in order to provide genetic evidence for our hypothesis that we had identified a rare gain-of-function allele in *ras85D* (Fig. 2). While searching for revertants, we also recovered several mutants that were strong suppressors of *GMR-hid*. Recognizing that these mutants might be synergizing with *ras1^R68Q^* to produce such a strong suppression, we successfully recovered and mapped 14 of these suppressors. As indicated in Table 1, most were mapped to a single candidate gene. Since these mutants were essentially derived from a dominant modifier screen for suppression of *GMR-hid* induced cell death, but within a sensitized *ras1^R68Q^* background, we expected the mutational spectrum to be overlapping, yet distinct from that of previous *GMR-hid* or *UAS-Ras^V12^* based screens. Indeed several suppressors turned out to overlap with ones identified previous screens. However, we also isolated two novel interactors: one allele of *notum* and four alleles of *Su(Tpl)*. This demonstrates the utility of *ras1^R68Q^* to identify novel genetic interactions. While *notum* affects the Wnt/Wingless signaling pathway, *Su(Tpl)* is thought to function in the regulation of transcription in response to stress [96], [97], [98].

Much of our understanding of Ras-mediated signaling is derived from a combination of biochemical experiments conducted in mammalian tissue culture, and genetic studies in model organisms [10]. For example, Ras-mediated signaling regulates the specification and differentiation of R7 photoreceptors in the *Drosophila* eye [80], [81], [99]. However, until now, studies on the physiological consequences of elevated Ras in *Drosophila* have relied on overexpression of the activated *ras1^v12^* allele [83], [85], [100]. The viable hypermorphic *ras1* allele described here, *ras1^R68Q^*, represents the first endogenous gain-of-function mutation in *Drosophila* Ras and hence offers a new tool for the analysis of Ras biology *in situ.* In particular, certain aspects of Ras biology have remained largely inaccessible to the use of constitutively active versions of this protein. This is because mutants, such as *ras1^v12^*, do not cycle normally between off and on states, are insensitive to regulatory circuits and are generally not compatible with organismal development. As a consequence, in certain paradigms and contexts, *ras1^v12^* actually behaves as a loss-of-function mutant rather than a hypermorph, occluding the biological interpretation of Ras function *in vivo* [101]. Therefore, the use of milder, viable hypermorphs of Ras, such as *ras1^R68Q^*, offers the potential for a refined understanding of the normal physiological roles of this important protein. Significantly, the *ras1^R68Q^* allele described here shares overall biochemical properties with recently discovered mutations in *k-ras* and *h-ras* that underlie human developmental disorders, such as Noonan, Costello and CFC syndromes.

## Supporting Information

Figure S1 **Genetic schemes for dominant modifier and reversion screens.** (A) *GMR-rpr* screen. *yw*; *GMR-rpr* ^81^ homozygous males were fed a solution of sucrose and 0.25mg/ml ENU or 25 mM EMS and mated to females of the same strain. F1 progeny were screened for suppression or enhancement of the parental rough eye phenotype. Of the 170,000 F1 progeny screened, ∼95% derived from ENU treated males, while 5% were from EMS treated males (B) *GMR-hid* screen. *yw* males were treated as above or with 4500 rad x-rays and then crossed to *GMR-hid* ^10^ homozygous females. F1 progeny were screened for suppression of the *GMR-*hid^10^ rough eye phenotype. Of the 300,000 F1 progeny screened, ∼49% derived from EMS treated males, ∼49% from x-ray treated males and 2% from ENU treated males. (C) Reversion screen. Homozygous *Su(21-3s)* males were treated with 4000 rad x-rays and crossed to *GMR-hid^1M^*; Sb/TM6B females. 80,000 F1 progeny were screened for loss of the *Su(21-3s)* suppression phenotype.
(TIF)

Figure S2 **The** ***Su(21-3s)*** **mutant differentially interacts with components of the EGFR/MAPK pathway.** Suppression of the *GMR-hid10* induced eye ablation phenotype by *Su(21-3s)* (A vs D) is not much affected by loss of function mutations in upstream components of MAPK signaling such as *egfr* (E) or *argos* (F), but is strongly ameliorated by loss of downstream components, such as *rolled* (B). Additionally, when a dominant negative form of Ras1 (*sev-ras1^N17^*) is expressed in the eye, the suppressive effects of *Su*(21-3s) are completely abrogated (C). Genotypes: (A) *GMR-hid^10^*/+, (B) *GMR-hid^10^*/rl^10A^;*Su(21-3s)*/+, (C) *GMR-hid^10^*/+;*Su(21-3s)*/*sev-ras1^N17^*, (D) *GMR-hid^10^*/+;*Su(21-3s)*/+, (E) *GMR-hid^10^*/*egfr* ^−^;*Su(21-3s)*/+, (F) *GMR-hid^10^*/+;*Su(21-3s)*/*arg^lΔ7^*.
(TIF)

Figure S3 **Overexpression of** ***ras1*** **in the wing induces ectopic vein material.** Overexpression of either wildtype *ras1* (C) or mutant *ras1^R68Q^* (D) using the *en-Gal4* driver results in the deposition of significant amounts of ectopic wing vein material. This phenotype is much more severe with *ras1^R68Q^* however, which frequently also results in wing blisters. Panels (A) and (B) are included for comparison only and are the same images shown in Figure 5.
(TIF)

Figure S4 **Overexpression of** ***ras1*** **in the eye induces developmental defects.** Both overgrowth and cell death phenotypes are observed when Ras is overexpressed in the fly eye. Flies overexpressing wildtype *ras1* (B,G) exhibit relatively minor disruptions in eye patterning and in the case of *sev-Gal4* driven expression, a small but significant amount of overgrowth occurs in the anterior part of the eye (G). In contrast, overexpression of *ras1^R68Q^* with *GMR-Gal4* (C-F) causes severe overgrowth and patterning disruptions. An example from each of four independent transgenic lines is shown to illustrate the range of phenotypes. Likewise, overexpression of *ras1^R68Q^* with *sev-Gal4* elicits a much more pronounced overgrowth phenotype in the anterior part of the eye (H) compared to that of wildtype *ras1* (G). Genotypes: (A) *GMR-Gal4*/+, (B) *GMR-Gal4*/+;UAS-*ras1*/+, (C-F) *GMR-Gal4*/+;UAS-*ras1^R68Q^*/+, (G) *sev-Gal4*/+;UAS-*ras1*/+, (H) *sev-Gal4*/+;UAS-*ras1^R68Q^*/+.
(TIF)

Table S1 ***GMR-rpr*** **modifiers: Summary of genetic interactions.** Complementation groups are named for the known gene to which they correspond. The group named “other” consists of mutants that could not be placed into complementation groups. *-th-st-* indicates that the mutation was roughly mapped by meiotic recombination around the markers *th* and *st* and may be located on either side, whereas *sr-e* indicates that the mutation maps between *sr* and *e*. Alleles with the same map position and similar phenotypes are grouped together for simplicity. Rep, reduced eye pigmentation; Sup, suppressor; Enh, enhancer; —, no effect; ND, not done.
(TIF)

Table S2 ***GMR-hid*** **suppressors: Summary of genetic interactions.** Legend is as described in Table S1. *-th-st-*, -*cu*- and -*sr*- indicate that the mutation was roughly mapped by meiotic recombination around the designated markers and may be located on either side, whereas *st-cu*, *cu-sr* and *sr-e* indicate that the mutation maps between the designated markers. The mutation characterized in this study, *Su(21-3s)*, is highlighted in yellow. Rep, reduced eye pigmentation; Ro, rough eye; Wv, extra wing veins; Wk, weak; Sup, suppressor; Enh, enhancer; -, no effect; ND, not done.
(TIF)
