# Supplementary figures and images for: A Gain-of-Function Germline Mutation in Drosophila ras1 Affects Apoptosis and Cell Fate during Development
Source: PLoS One. 2011 Aug 12;6(8):e23535. doi: 10.1371/journal.pone.0023535 (PMC3155559; doi:10.1371/journal.pone.0023535)

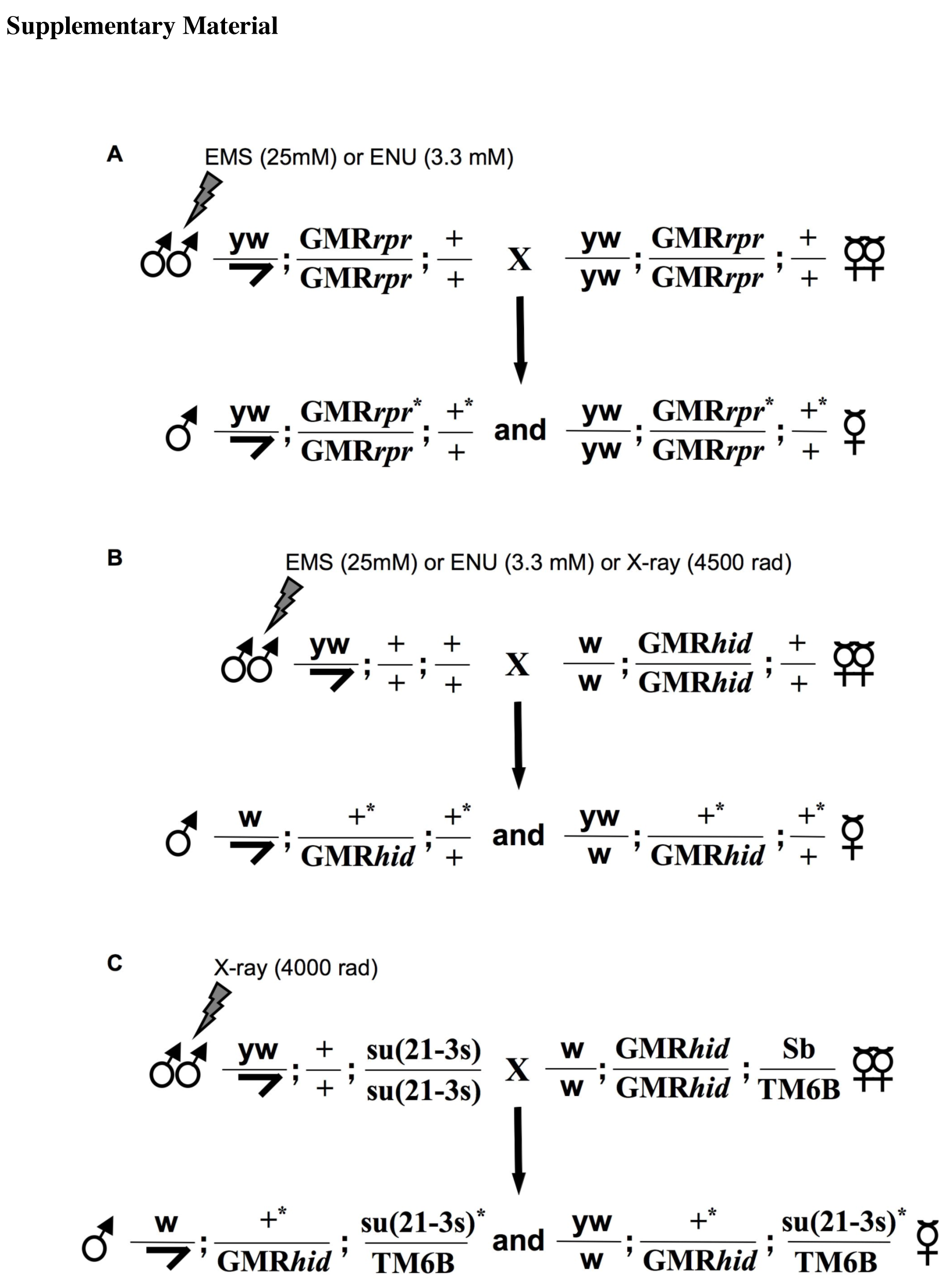

Supplement: Figure S1 — Genetic schemes for dominant modifier and reversion screens. (A) GMR-rpr screen. yw; GMR-rpr 81 homozygous males were fed a solution of sucrose and 0.25mg/ml ENU or 25 mM EMS and mated to females of the same strain. F1 progeny were screened for suppression or enhancement of the parental rough eye phenotype. Of the 170,000 F1 progeny screened, ∼95% derived from ENU treated males, while 5% were from EMS treated males (B) GMR-hid screen. yw males were treated as above or with 4500 rad x-rays and then crossed to GMR-hid 10 homozygous females. F1 progeny were screened for suppression of the GMR-hid10 rough eye phenotype. Of the 300,000 F1 progeny screened, ∼49% derived from EMS treated males, ∼49% from x-ray treated males and 2% from ENU treated males. (C) Reversion screen. Homozygous Su(21-3s) males were treated with 4000 rad x-rays and crossed to GMR-hid1M; Sb/TM6B females. 80,000 F1 progeny were screened for loss of the Su(21-3s) suppression phenotype. (TIF) [file pone.0023535.s001.tif]

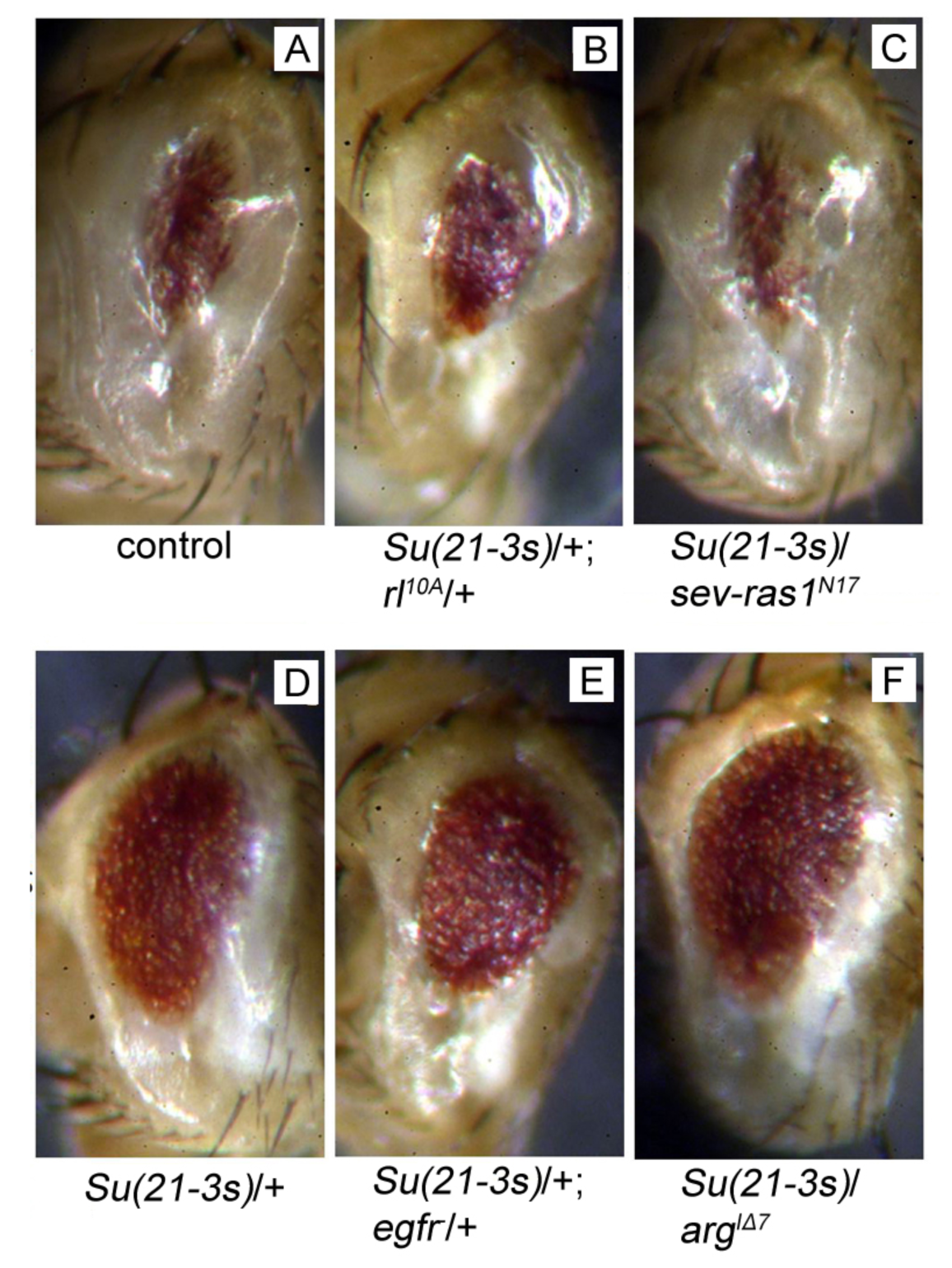

Supplement: Figure S2 — The Su(21-3s) mutant differentially interacts with components of the EGFR/MAPK pathway. Suppression of the GMR-hid10 induced eye ablation phenotype by Su(21-3s) (A vs D) is not much affected by loss of function mutations in upstream components of MAPK signaling such as egfr (E) or argos (F), but is strongly ameliorated by loss of downstream components, such as rolled (B). Additionally, when a dominant negative form of Ras1 (sev-ras1N17) is expressed in the eye, the suppressive effects of Su(21-3s) are completely abrogated (C). Genotypes: (A) GMR-hid10/+, (B) GMR-hid10/rl10A;Su(21-3s)/+, (C) GMR-hid10/+;Su(21-3s)/sev-ras1N17, (D) GMR-hid10/+;Su(21-3s)/+, (E) GMR-hid10/egfr −;Su(21-3s)/+, (F) GMR-hid10/+;Su(21-3s)/arglΔ7. (TIF) [file pone.0023535.s002.tif]

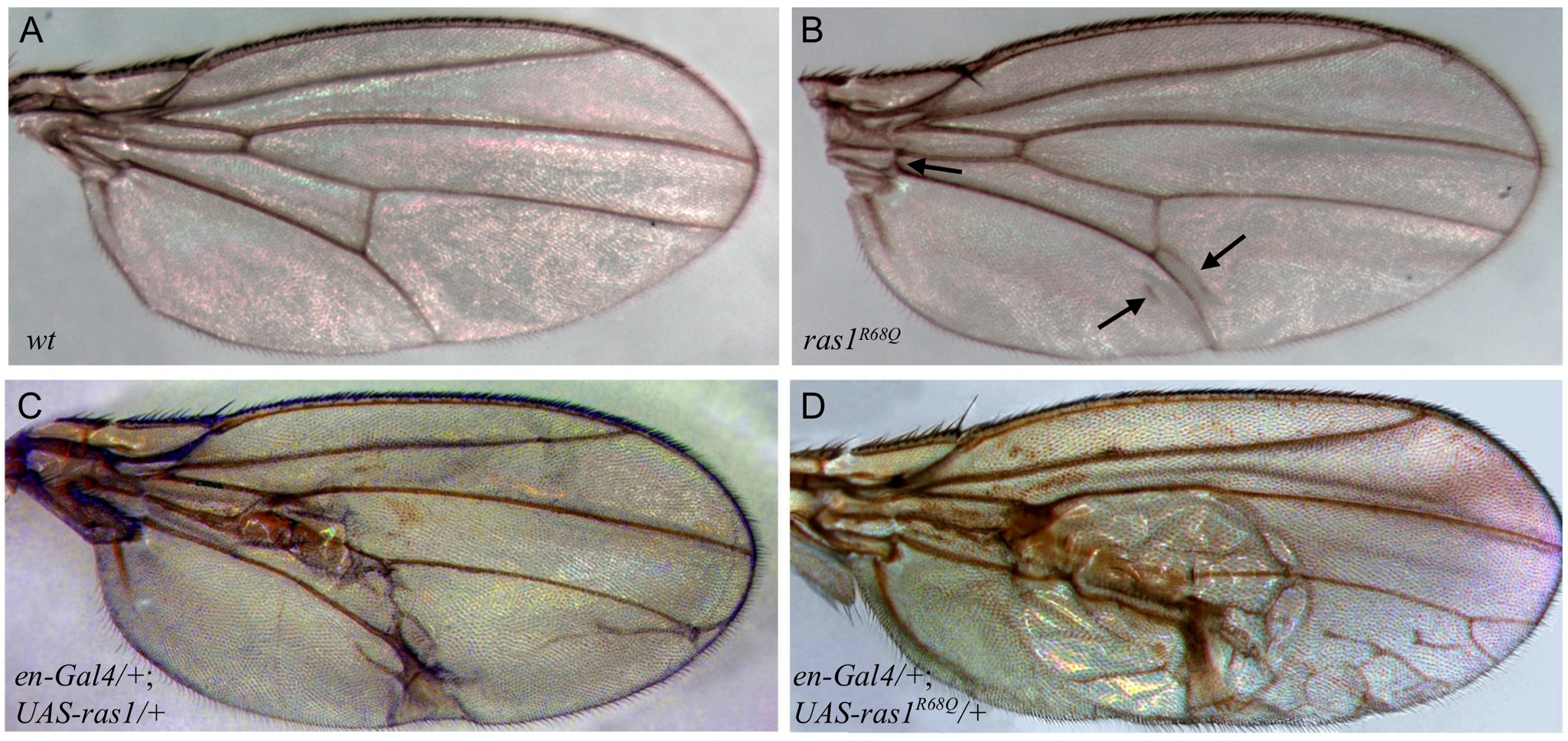

Supplement: Figure S3 — Overexpression of ras1 in the wing induces ectopic vein material. Overexpression of either wildtype ras1 (C) or mutant ras1R68Q (D) using the en-Gal4 driver results in the deposition of significant amounts of ectopic wing vein material. This phenotype is much more severe with ras1R68Q however, which frequently also results in wing blisters. Panels (A) and (B) are included for comparison only and are the same images shown in Figure 5. (TIF) [file pone.0023535.s003.tif]

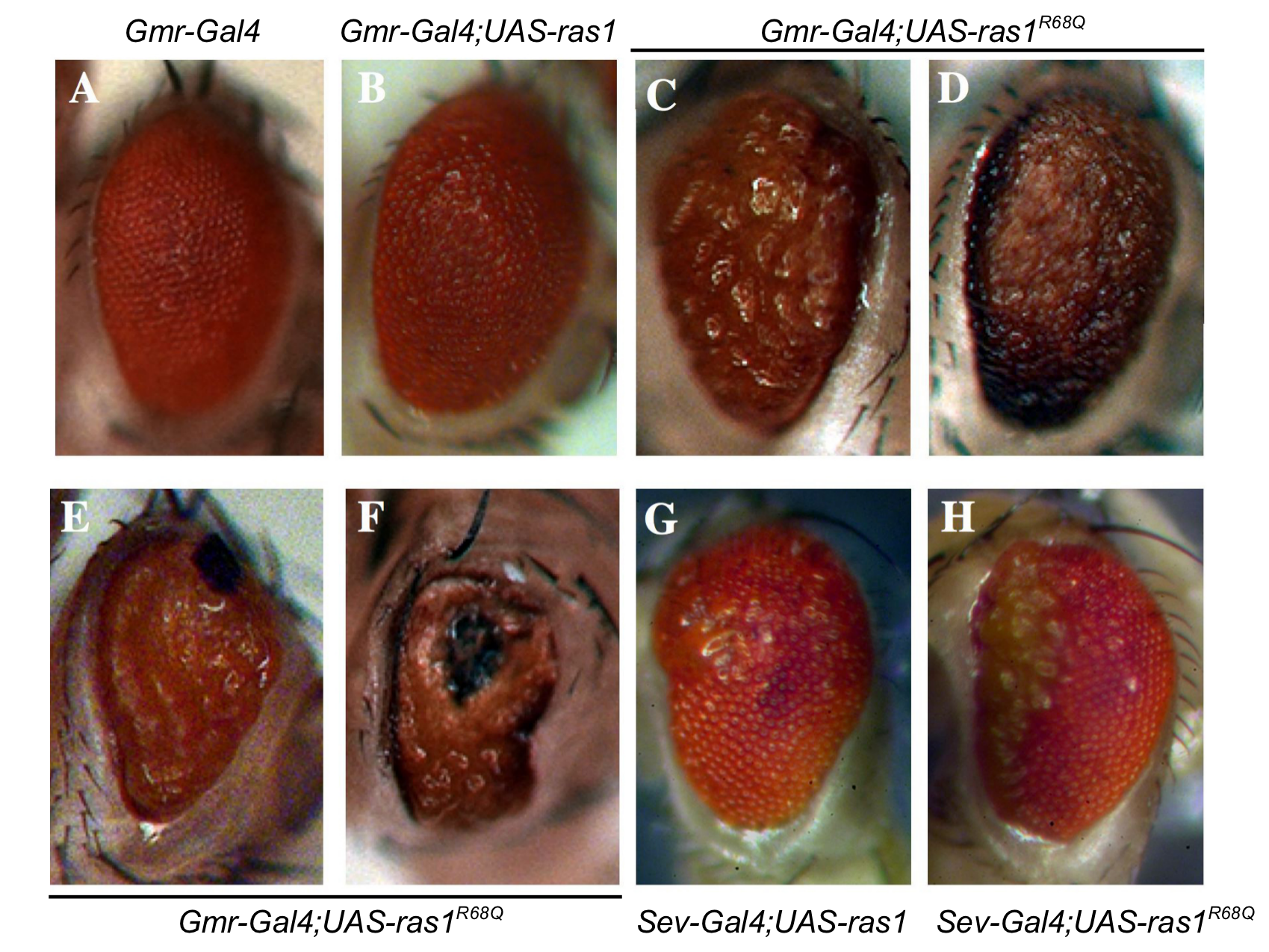

Supplement: Figure S4 — Overexpression of ras1 in the eye induces developmental defects. Both overgrowth and cell death phenotypes are observed when Ras is overexpressed in the fly eye. Flies overexpressing wildtype ras1 (B,G) exhibit relatively minor disruptions in eye patterning and in the case of sev-Gal4 driven expression, a small but significant amount of overgrowth occurs in the anterior part of the eye (G). In contrast, overexpression of ras1R68Q with GMR-Gal4 (C-F) causes severe overgrowth and patterning disruptions. An example from each of four independent transgenic lines is shown to illustrate the range of phenotypes. Likewise, overexpression of ras1R68Q with sev-Gal4 elicits a much more pronounced overgrowth phenotype in the anterior part of the eye (H) compared to that of wildtype ras1 (G). Genotypes: (A) GMR-Gal4/+, (B) GMR-Gal4/+;UAS-ras1/+, (C-F) GMR-Gal4/+;UAS-ras1R68Q/+, (G) sev-Gal4/+;UAS-ras1/+, (H) sev-Gal4/+;UAS-ras1R68Q/+. (TIF) [file pone.0023535.s004.tif]

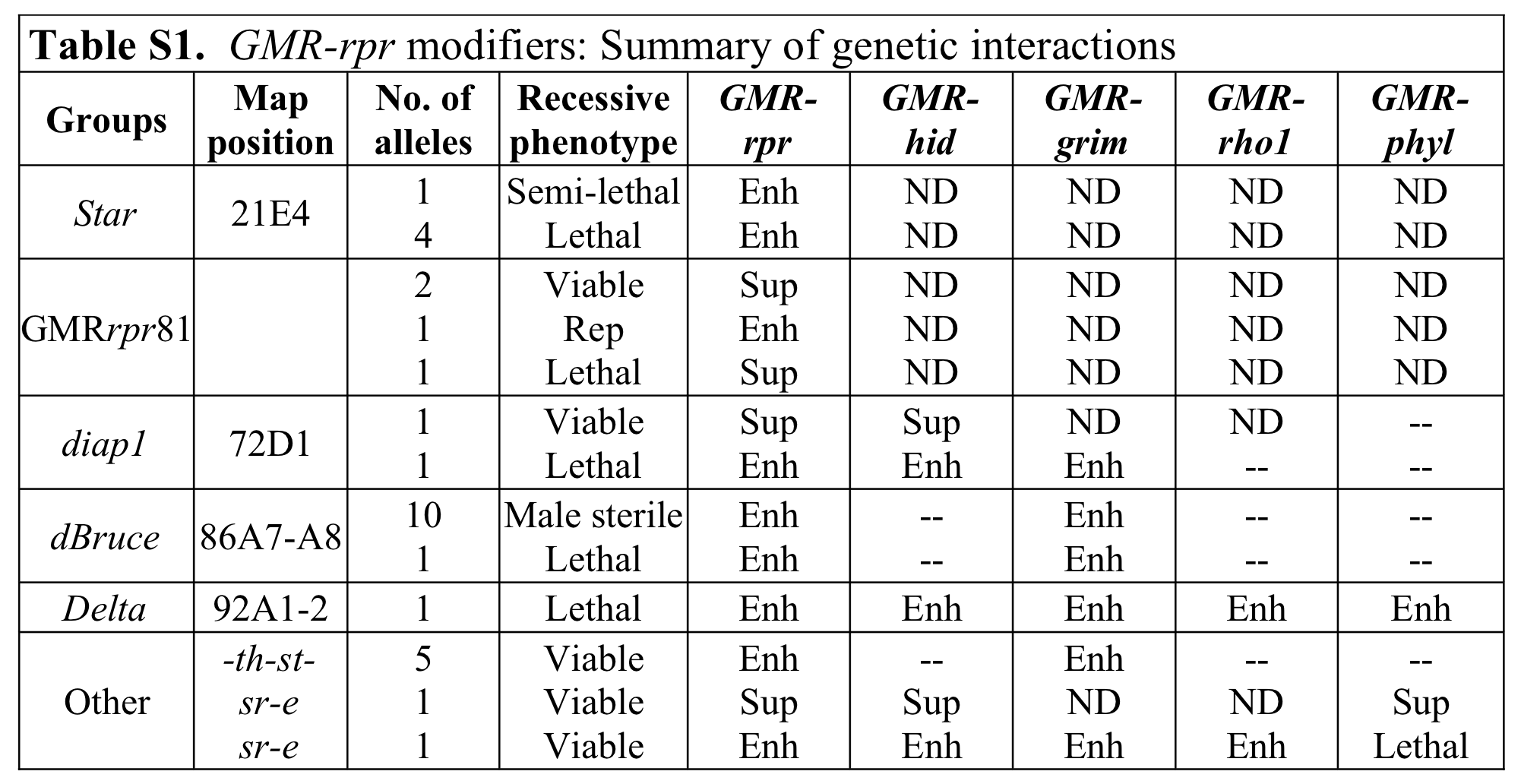

Supplement: Table S1 — GMR-rpr modifiers: Summary of genetic interactions. Complementation groups are named for the known gene to which they correspond. The group named “other” consists of mutants that could not be placed into complementation groups. -th-st- indicates that the mutation was roughly mapped by meiotic recombination around the markers th and st and may be located on either side, whereas sr-e indicates that the mutation maps between sr and e. Alleles with the same map position and similar phenotypes are grouped together for simplicity. Rep, reduced eye pigmentation; Sup, suppressor; Enh, enhancer; —, no effect; ND, not done. (TIF) [file pone.0023535.s005.tif]

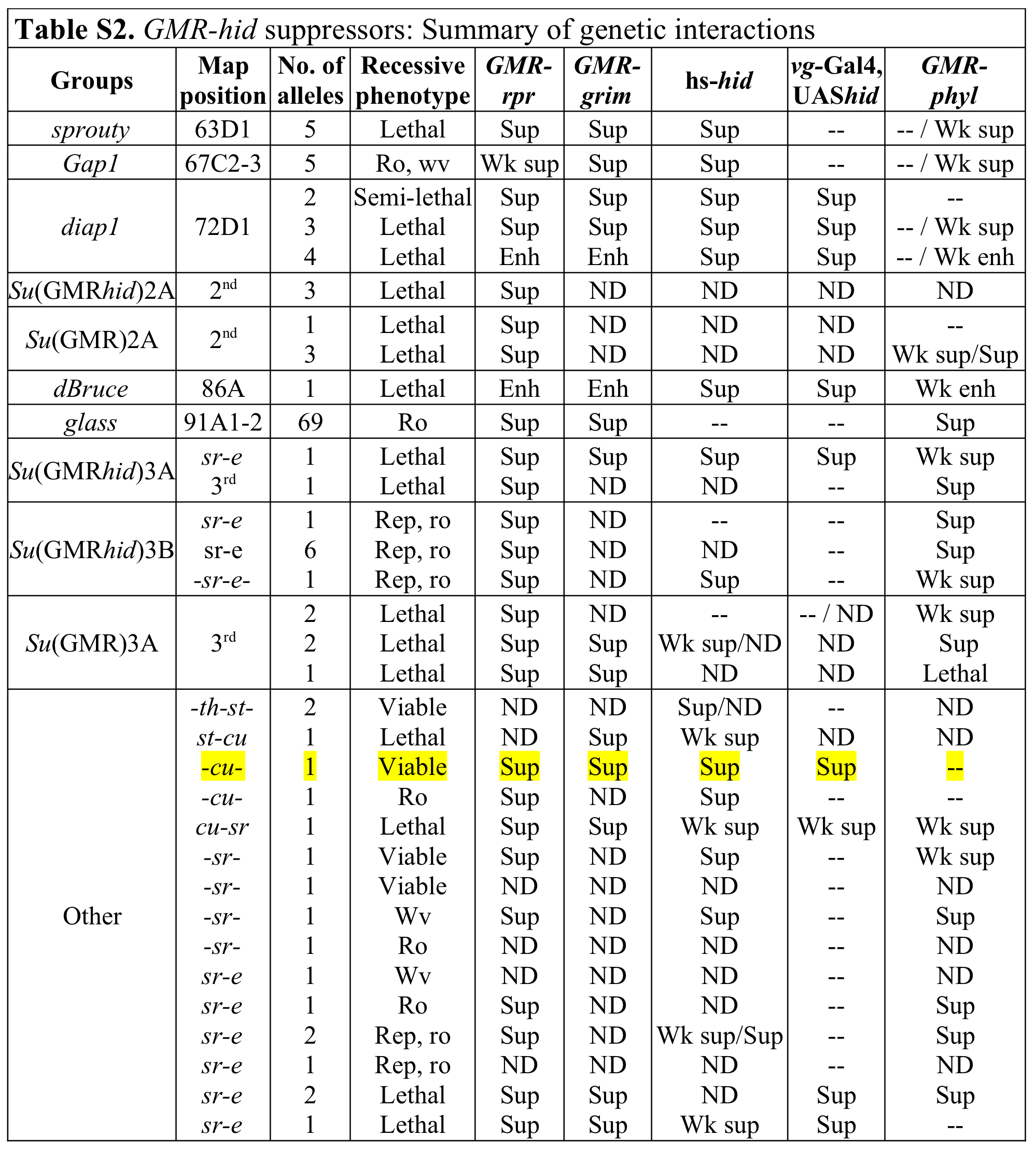

Supplement: Table S2 — GMR-hid suppressors: Summary of genetic interactions. Legend is as described in Table S1. -th-st-, -cu- and -sr- indicate that the mutation was roughly mapped by meiotic recombination around the designated markers and may be located on either side, whereas st-cu, cu-sr and sr-e indicate that the mutation maps between the designated markers. The mutation characterized in this study, Su(21-3s), is highlighted in yellow. Rep, reduced eye pigmentation; Ro, rough eye; Wv, extra wing veins; Wk, weak; Sup, suppressor; Enh, enhancer; -, no effect; ND, not done. (TIF) [file pone.0023535.s006.tif]
